# Dimensional Stability of Beech Wood: The Influence of Taper, Slope of Annual Ring and Sawing Pattern

**DOI:** 10.3390/polym17233158

**Published:** 2025-11-27

**Authors:** Peter Vilkovský, Tatiana Vilkovská, Ivan Klement, Martin Fúčela

**Affiliations:** 1 Faculty of Wood Sciences and Technology, Department of Wood Technology, Technical University in Zvolen, T. G. Masaryka 24, 96001 Zvolen, Slovakia; tatiana.vilkovska@tuzvo.sk (T.V.); klement@tuzvo.sk (I.K.); 2 Faculty of Wood Sciences and Technology, Department of Wooden Constructions, Technical University in Zvolen, T. G. Masaryka 24, 96001 Zvolen, Slovakia; martinfucela@gmail.com

**Keywords:** beech, sawing pattern, shape stability, slope of annual ring, warps

## Abstract

The dimensional stability of sawn timber is one of the key factors affecting processing and final application in various fields, such as construction, furniture making, and interior design. One of the most common problems that beech wood producers may confront is the occurrence of various types of warping (deformation) during drying. These warps significantly affect the processability of sawn timber, which can lead to reduced yield and economic losses. Several factors can affect dimensional stability. These factors include the sawing pattern, the position of the timber in the log, and the slope of the annual rings. Our research investigated these factors and focused on two types of warping: cup and twist. The results showed a notable influence of the original position of the timber in the log on the degree of cup warping after drying (r = 0.5194; *p* = 0.0189), with timber closer to the perimeter exhibiting less curvature. The sawing pattern (parallel to the surface of the log—RsP; parallel to the axis of the log—RsO) had a less significant effect but showed a tendency towards curvature (r = 0.4242; *p* = 0.0623). Based on the sawing pattern, after drying, the twist warping was more pronounced in RsP logs, while RsO cuts retained better shape stability and had only minimal cup warping.

## 1. Introduction

Beech is one of the most widely used types of wood. Due to its properties, it is used in the manufacture of floors, stairs, furniture, musical instruments, kitchen utensils, agricultural tools, and craft tools. In recent years, the development and use of beech wood in construction have also increased [1]. European beech (*Fagus sylvatica* L.) is a tree species with a high frequency of defects that significantly affect its quality. Many defects (such as red fault heartwood, knots, and cracks) can be easily identified during the processing of raw beech wood and can be easily eliminated. However, some defects can only be identified during sawing or due to negative effects during further processing [2,3].

When processing logs, it is very common to find taper, which we understand as the longitudinal narrowing of the log from a thicker diameter to a thinner diameter [4,5]. Also, [6] characterizes taper as the slope of the outer line derived from diameters measured along the entire length of the tree. This parameter is often basic geometric data that affects its further processing.

In their research, refs. [7,8] investigated the impact of taper on further processing and found that the greater the degree of taper, the worse the volume and quality of the lumber yield. Ref. [8] evaluated the taper and its impact on the future strength and dimensional stability of future timber.

Wood shrinkage is a process in which the dimensions of wood decrease as a result of the loss of bound water from the wood. Wood shrinkage is anisotropic in character, as evidenced by different shrinkage values in different anatomical directions. The greater the difference between radial and tangential moisture movement, the more pronounced the shape changes [9]. Growth stresses and their expression are closely related to the quality or dimensional stability of future sawn timber. In the research, ref. [10] investigated the influence of the distribution of internal stresses in the logs on the warping of sawn timber after sawing, with attention to the connection between the annual ring inclination and the resulting shape changes. Using numerical simulation based on bend theory, the authors found that the loss of symmetry of internal stresses with respect to the annual ring inclination leads to more pronounced warping, especially bowing and twisting. If the annual rings are arranged symmetrically, i.e., perpendicular to the surface, the lumber remains more stable in shape. Conversely, if the annual rings are asymmetrical or slanted, the release of internal stresses leads to warping and a deterioration in shape stability. The authors emphasized that the geometry of the cross-section and the inclination of the annual rings are key factors that, in combination with the distribution of stresses, determine the resulting stability of the timber after the sawn process. According to [11], the slope of the annual ring has a remarkable effect on the final shape stability of sawn timber. The authors analyzed the warping of sawn timber with different slopes of annual rings after sawing and drying using numerical simulation. The results showed that the slope of annual rings is closely related to the degree of twist and bow of sawn timber. Sawn timber with a slope of annual rings typical for sawn timber from the area near the pith end tended to have greater twist warping. In contrast, timbers with annual rings whose slope of inclination was close to 90° showed the highest dimensional stability and exhibited virtually no warping. The authors also noted that tangential drying in combination with the inclination of annual rings causes irregular stresses that lead to the formation of warps during drying. From the point of view of dimensional stability, it is therefore advantageous for the annual rings to be laid at a slope of 70 to 90°, thus minimizing internal stresses and warping during drying. In his work, ref. [12] focused on the numerical analysis of deformations in sawn timber caused by changes in moisture content during drying and processing. Using a three-dimensional model based on the method of finite elements, he examined how differences in the orthotropic properties of wood, the slope of annual rings, the twist of fibers, and internal growth stresses affect the formation of shape deformations such as spiral, transverse, and other types of warping. The results showed that a significant amount of this warping occurs already when the logs are sawn due to the release of growth stresses, while others develop during drying depending on the anisotropy and position of the sawn timber in the log.

The original position timber in the log is another important parameter affecting the future dimensional stability of sawn timber. A similar observation was also found in an article written by [13], where the authors focused on determining the effect of the position of the pith on the warping of sawn timber after the drying process. The study focused on medium-quality logs and monitored how the central versus eccentric pith changes the degree of warping of the timber. The results showed that timber produced from logs with an eccentric pith tends to warp more, which can negatively affect its workability and quality as well.

Different methods of sawing patterns of logs can significantly affect the yield and quality of lumber. This issue has been addressed by several authors, including [10,13,14]. The research by [14] focused on comparing different sawing patterns in the processing of large Sugi (*Cryptomeria japonica*) sawn wood and analyzed their impact on yield and quality, including the presence of knots, cracks, and warping. The findings showed that choosing a suitable sawing pattern can reduce waste, maximize economic yield, and ensure higher board quality, which is crucial for the efficient and sustainable processing of large-diameter logs.

According to [15], beech is one of the wood species for which the issue of dimensional stability is particularly important for future processing.

Therefore, our research aimed to analyze the influence of the sawing method and the original position of the timber in the log on its dimensional stability of beech after the drying process, focusing on the transverse (cup) and twist warping of timber. The result is to contribute to the optimization of beech log processing into timber and improve the quality of the final products as well.

## 2. Materials and Methods

The selection of European beech (*Fagus sylvatica* L.) samples for the purposes of this research was carried out on the basis of predetermined conditions. The cut had to have a diameter of more than 50 cm at the thinner end, without any evidence of warping, eccentric pith, or evidence of biological degradation of the red false heartwood and sapwood. The four selected pieces of logs were then shortened to a length of 4 m. They were measured and processed in the Technical University of Zvolen (Slovakia) workshops and laboratories.

### 2.1. Determining the Taper of the Logs

We understand the taper of the logs as the degree to which the cross-section of the logs decreases or narrows from the thicker end towards the thinner end. For this work, it was necessary to determine this data from the dimensional characteristics of the initial raw material (*Fagus sylvatica* L.). The basic data required to calculate the taper were the length of the cut, the diameter at the thinner end of the log (d), the diameter at the thicker end of the log (D), and the length of the log. It was determined in cm per 1 m of cut length. All these data were evaluated based on calculations according to the study by [5].

(1) $$\mathrm{Taper}=\left(\frac{D_{1}+D_{2}}{2}\right)-\left(\frac{d_{1}+d_{2}}{2}\right)/L\;\;[\text{cm}/\text{m}]$$

where *d*_1_—diameter at the thinner end in cm (thicker diameter), *d*_2_—diameter at the thinner end in cm (thinner diameter), *D*_1_—diameter at the thicker end in cm (thicker diameter), *D*_2_—diameter at the thicker end in cm (thinner diameter), and *L*—length of log.

### 2.2. Sawn Pattern of Beech Logs

**Figure 1:**
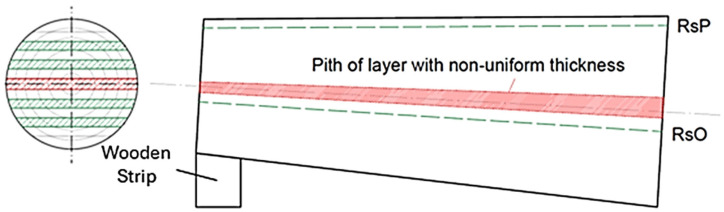
Designed sawing pattern using the RsP and RsO sawing methods.

A sawing pattern through and through (live pattern) was designed for the logs (Figure 1), which was carried out in two stages. In the first stage (first half of the log), the saw was parallel to the surface of the log (RsP), while in the second stage (second half of the log), the saw was parallel to the axis of the log (RsO). The correct position of the log was set using stickers with different thicknesses (the thickness of the stickers depended on the taper of the log).

As a result of the change in the type of sawing pattern between the first and second stages, a timber with asymmetrical thickness at each end was created in the central part of the log (Figure 1, marked in red). This unedged sawn timber layer contained a large proportion of pith, which could significantly affect the dimensional stability of the future edged timber. For these reasons, this layer was removed. The sawing pattern was performed on a Mebor HTZ 1000 horizontal log band saw. The thickness of the future timber was 32 mm in the fresh state.

**Figure 2:**
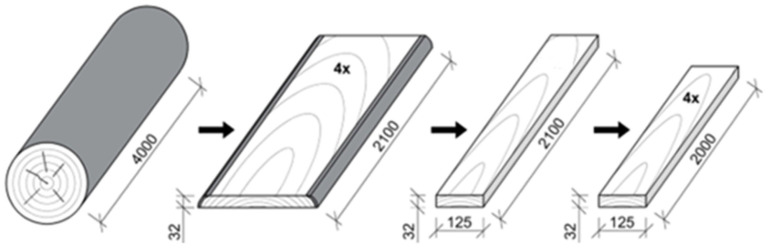
Graphical representation of the production process of beech-edged timber.

Every second layer was selected from each log for evaluation of the effect of annual ring slope (Figure 1), resulting in a total of 16 pieces of unedged timber. This timber was then edged and sawn to a width of 125 mm (fresh state) on an SCM Nova SI300s circular saw and shortened to a length of 2100 mm (Figure 2). After sawing, 16 boards were selected for further processing. The rest was used to fill the timber pile. The selected lumber (16 pieces) was trimmed at the ends and shortened to a length of 2000 mm.

### 2.3. Measuring the Slope of Annual Rings

**Figure 3:**
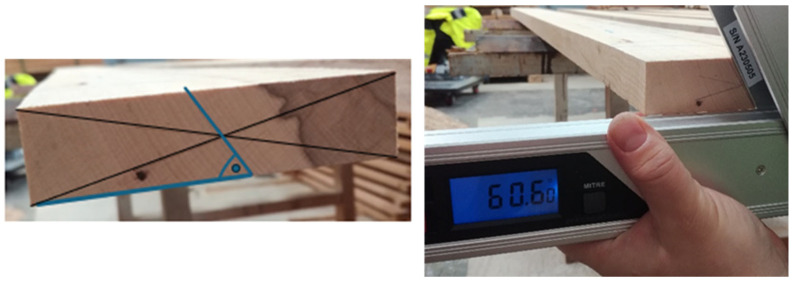
Procedure for measuring the slope of annual rings using a digital protractor.

To correctly classify the sawn timber into individual categories (tangential, radial), we had to determine the slope of the annual rings using a digital protractor—Type 0-42-087 (Measuring range from 0° to 225° (angle), Accuracy: ±0.1°). This procedure was applied to 16 selected samples on both ends. The procedure is shown in Figure 3 [16]. Based on these values, the lumber could then be classified as radial (60–90°) or tangential timber (0–30°).

### 2.4. Stacking the Timber Pile

**Figure 4:**
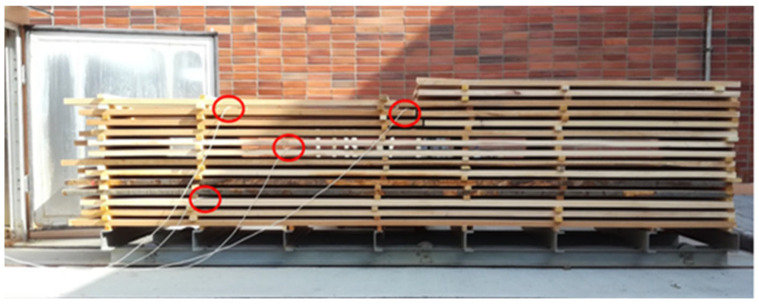
Drying pile with marked positions of sensors for measuring moisture content (red circle) during drying.

Drying was carried out in a drying kiln from KATRES (type KS MINI 4.5 with electrical power input of 3 kW). The drying pile was created from sawn timber stacked in regular layers separated by strips with a cross-section dimension of 35 × 20 mm. The pile itself consisted of 13 layers (Figure 4). At the top of the pile were two layers with samples intended for measuring dimensional changes during drying.

Artificial drying was carried out in a chamber dryer using a standard schedule based on beech wood and a thickness of 32 mm. The moisture content was reduced from 60% to 14 ± 2%. The process stages lasted as follows: heating 16.75 h, drying 283.5 h, equalization 6.5 h, conditioning 6.25 h, and cooling 3.25 h.

### 2.5. Measuring Shape Changes Using ARAMIS

Digital image correlation (DIC) is an optical method for measuring displacements and relative deformations of objects using digital surface scanning. The starting point is a reference image (before curvature), which is compared with images after curvature (e.g., after drying the sample). The surface of the sample is covered with a colored pattern and divided into small sub-areas. The system compares their position before and after warping, thereby determining the movement of individual points [17].

We separate plane correlation (one camera) and space analysis, where a stereoscopic camera setup is used [17]. The three-dimensional analysis we used allows us to obtain 3D coordinates of points using the ARAMIS non-contact optical measurement system. It records surface structures, assigns coordinates to pixels, and determines the degree of warping based on a comparison of images.

Our system consisted of two high-speed cameras (12 Mpx, depth of field 25 mm) and two external lights. The size of the grid was 19 × 19 pixels with a 2-pixel overlap.

**Figure 5:**
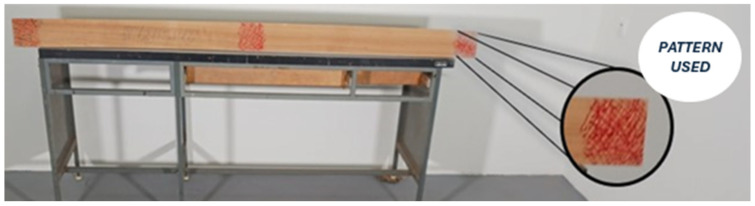
Example of a sawing pattern used when scanning with high-speed cameras.

The character of the surface of the measured object is crucial when carrying out measurements. The surface of the measured object must be marked with a pattern so that the pixels in the camera image can be clearly assigned. The pattern we chose consisted of a network of random lines and dots (Figure 5). We applied this pattern to all samples on both edges and the center of the board as well.

The patterns were drawn with markers in four colors according to the log from which they were manipulated: blue = log I; black = log II; green = log III; red = log IV. The measurements were taken after selecting samples from the drying room, approximately every third day, in a laboratory at the Technical University in Zvolen under constant lighting conditions and an average temperature of 16.7 °C.

## 3. Results and Discussion

### 3.1. Evaluation of Log Taper

Log taper is one of the key factors affecting mechanical properties and dimensional stability, so its accurate evaluation is essential to ensure the future quality of sawn timber. The calculated values of log taper (Table 1) showed that it did not reach values that could affect the future stability of the sawn timber. Also, when compared with another author (Table 1), it is clear that our logs did not exceed the taper risk values. The final evaluation shows that our logs had normal taper, meaning they should have a minimal impact on the stability of the timber or the appearance of the observed types of warping.

Based on the study, ref. [8] found that beech logs with less taper (i.e., with a more uniform diameter along their length) have better mechanical properties and probably also better lumber stability. Similarly, research by [10] on the influence of log shape on internal stress distribution confirmed that logs with greater taper show greater differences in stress, which can manifest itself in the form of more severe warping of the timber. In their research, ref. [3] analyzed the effect of the method of sawing beech logs on the yield and longitudinal warping of timber. The authors found that for logs with a taper of ≥1.3 cm·m^−1^, it is more beneficial to use sawing parallel to the log axis (RsO), which leads to higher yield and less longitudinal warping. Similarly, study [7] showed that logs exhibiting lower taper and larger diameters achieve higher yields. Logs with greater taper produced more waste and were prone to higher longitudinal deformation. Their results emphasize that selecting logs with lower taper and optimizing the sawing method are key factors for improving both the efficiency and quality of lumber production. Sawing pattern for sharp-edged timber

**Figure 6:**
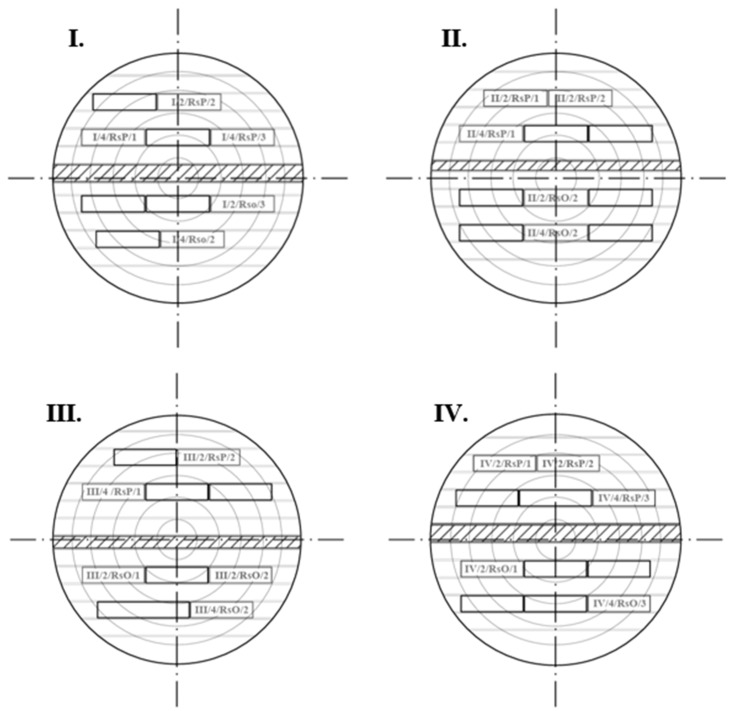
Sawing pattern used for individual logs with sample positions in the stem.

Sawing was performed using a standard through-and-through sawing pattern. The final sawing patterns are shown in Figure 6, with marked samples and their positions. From cuts I, II, and IV, we obtained 5 pieces of unedged timber from both stages of sawing. For log III, there were 6 pieces from the first stage and 5 from the second stage.

**Figure 7:**
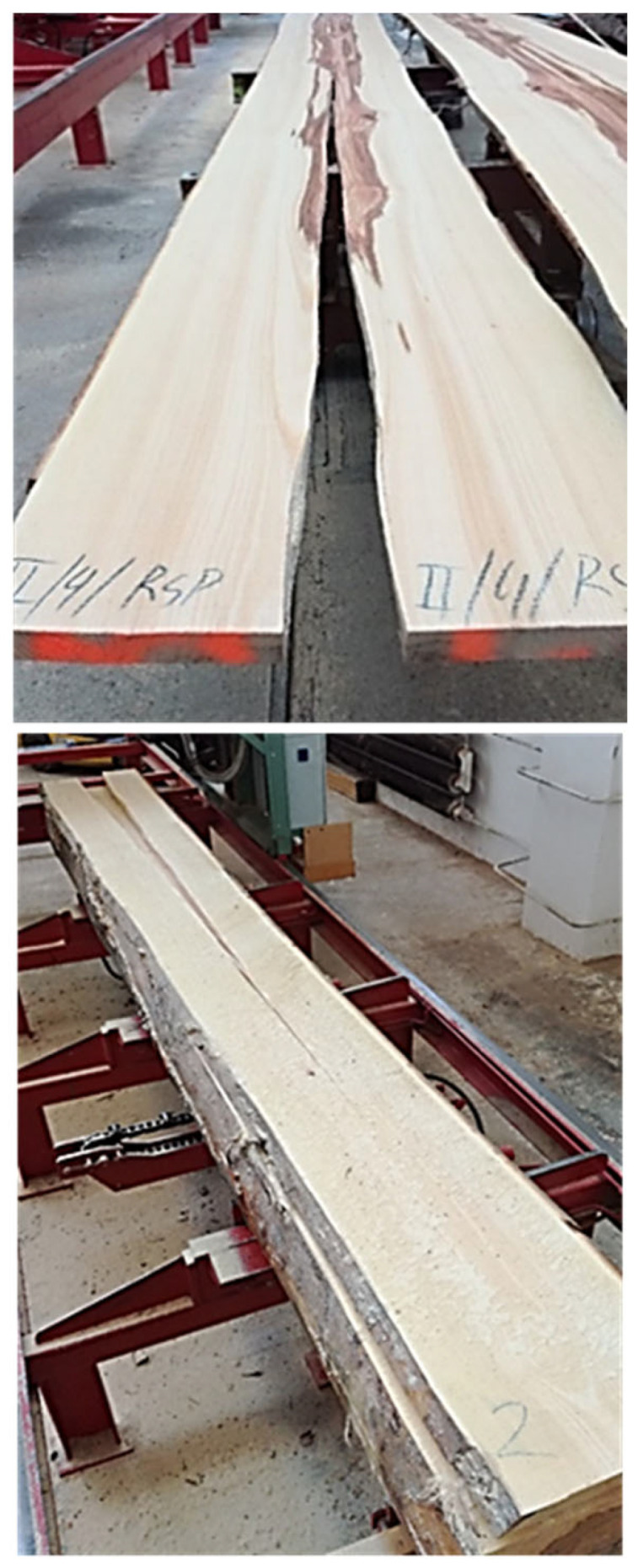
Example of cracks in the second layer of section II (top image) and the fourth layer of section II (bottom image).

For cut II, layers 2 to 5 cracked immediately after sawing in the first stage (sawing parallel to the surface). Similarly, layers 3 to 5 cracked for log IV. This was probably caused by high levels of internal stress due to the tree’s growth conditions (Figure 7). Since we wanted to determine the dimensional stability of beech timber without the effect of the pith, it was removed when changing the log (from RSP to RsO). Only the area outside or near the perimeter of the log was evaluated. During cutting, an immediate manifestation of longitudinal surface warping (bow) was identified, mainly in log II. This was probably caused by the uneven distribution of growth stresses, which led to immediate warping after the sawing process. Also, log III had a taper of 1.35 cm/m, which could have affected the distribution of growth stresses and the appearance of warping to some magnitude. This is confirmed by research by [10], who similarly found that logs with greater taper are more prone to greater non-uniformity in the distribution of growth stresses, which can be reflected in more pronounced warping of the timber. Even added that these warping can also be affected by various other factors, such as fiber twist, slope of annual ring, etc.

Our results also showed that RsP cutting resulted in greater growth stresses and more significant warping, mainly of the twisted type. This claim can also be supported by the fact that this method resulted in the cutting of fibers, which could have resulted in more significant warping (twist). According to a study by [14], different sawing methods can lead to uneven distribution of growth stresses in sawn timber, which has a direct impact on its dimensional stability. The use of a suitable sawing pattern can thus significantly contribute to better lumber quality, not only by reducing dimensional instability, but also by increasing the strength properties of the timber as well.

Based on the study [1], one of the main technical challenges in using European beech wood is its lower dimensional stability compared to some coniferous species, such as Norway spruce (*Picea abies*). This issue is particularly evident in multi-layered beech products, for example, laminated veneer lumber (LVL) made from beech shows higher swelling and shrinkage coefficients when exposed to changes in moisture content. The tangential shrinkage of beech wood is reported to be 12.3 %, highlighting its susceptibility to dimensional changes. Such properties can affect its suitability for structural applications, where dimensional stability and predictable behavior under varying moisture content are crucial.

### 3.2. The Effect of Annual Ring Slope on the Dimensional Stability of Beech Lumber

The slope of annual rings directly affects the shrinkage and swelling of wood, which has a direct impact on the dimensional stability/instability of the sawn timber. In our research, we therefore focused on evaluating the influence of the inclination of annual rings on the dimensional stability of beech timber. The timber was sawn from different positions in the log, which gave us samples that differed, among other things, in the inclination of the annual rings. It is clear that with this method of sawing, the layers (of the required timber thickness) were gradually separated from the surface of the log to its center, and the log sawing was subsequently changed. This procedure allowed us to create timber sawn parallel to the surface (RsP) and then parallel to the axis (RsO) of the log. After measuring the slope of the annual rings (Table 2), we determined the proportion of timber with a smaller (tangential timber) and larger (radial timber) slope of the annual rings. The proportion ranged from 60% tangential timber to 40% radial timber. Table 2 also shows the values of transverse (cup) and twist warping. The results show that timber with an annual ring slope of 0° to 35.5° exhibited higher values of both twist and cup warping. The average values for spiral warping and tangential sawn timber were 18.40 mm compared to radial sawn timber, which had 9.27 mm (50% lower warp). For cup warping, the trend was similar for tangential sawn timber at 1.06 mm and almost 50% lower for radial sawn timber at 0.66 mm. The research thus showed that the slope of annual rings significantly affects the dimensional stability of beech sawn timber. It also points out that timber with an annual ring inclination of 60° to 90° appears to be more dimensionally stable, while timber with an annual ring inclination of 0° to 40° has a greater tendency towards dimensional instability (increase in transverse (cup) and twist warping).

According to [10,11], the slope of annual rings has a significant impact on the shape stability of sawn timber. Numerical simulations in both studies showed that as the slope of annual rings increases, the tendency to warp, especially twist and bow (spring), also increases. Lumber from areas close to the pith, where the annual rings are significantly warped (0 to 30°), showed a higher degree of twist, while boards with annual rings perpendicular to the surface (70 to 90°) remained more dimensionally stable. The results also confirmed that the asymmetrical arrangement of annual rings disrupts the balance of internal stresses in the cross-section, leading to warping during drying. In terms of quality and dimensional stability, it is therefore advantageous for the annual rings to be as perpendicular to the surface of the timber as possible.

**Figure 8:**
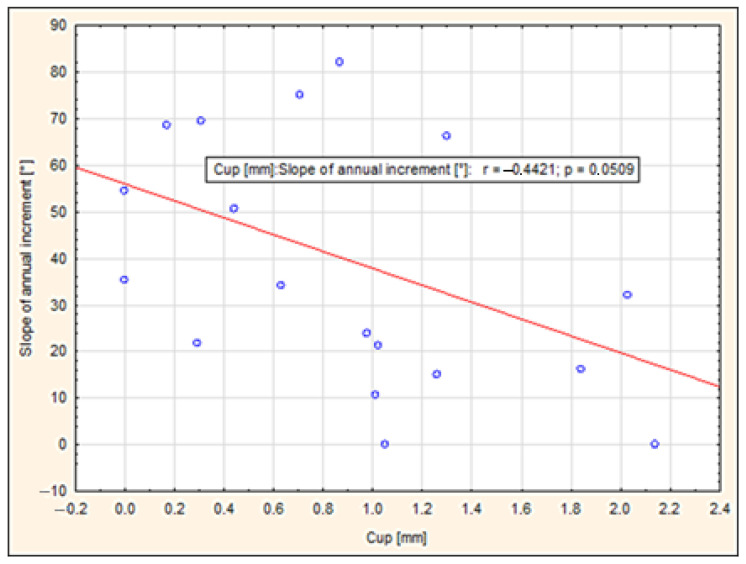
Dependence of transverse warping on the slope of annual rings.

Figure 8 shows the relationship between the slope of annual rings and the value of transverse warping. Correlation analysis showed a moderately strong negative correlation (r = −0.4421), which means that as the slope of annual rings increases, transverse warping decreases. This trend is also confirmed by the regression line, which has a downward slope. The significance level *p* = 0.0509 is just above the 0.05 threshold, indicating that the relationship is on the verge of statistical significance. The results, therefore, indicate that timber with a higher slope of annual rings tends to exhibit better dimensional stability, but a larger sample set would be needed for definitive confirmation.

Correlation analysis for twist warping showed a negative correlation (r = −0.3233), indicating that the twist warping of timber decreases with increasing annual ring slope. Despite this trend, the dependence was not statistically significant (*p* = 0.1644) and therefore cannot be considered unequivocally confirmed. The variance of the measured values was significant, indicating that factors other than the slope of the annual rings themselves also influence the resulting amount of twist.

### 3.3. The Influence of the Sawing Pattern on the Dimensional Stability of Beech Timber

In practice, the sawing pattern has a significant influence on the future dimensional stability of the timber. A correctly selected sawing pattern helps to prevent the timber from warping and ensures that the product retains its desired shape in the long term.

**Figure 9:**
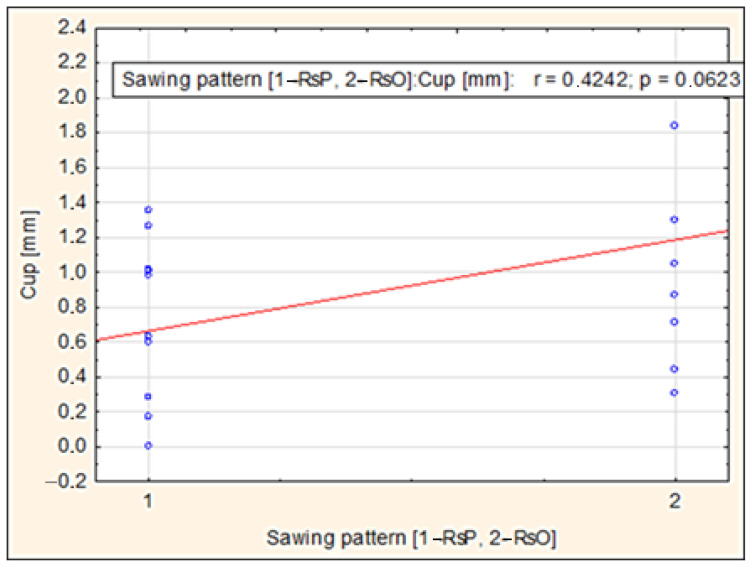
Dependence of cup warping on the sawing pattern (RsP and RsO).

Figure 9 shows the relationship between the sawing pattern (RsP, RsO) and the value of transverse warping (Cup) in mm. The correlation analysis shows that there is a moderately strong positive relationship between these variables (r = 0.4242), i.e., when switching from pattern RsP (1) to RsO (2), the curvature tends to increase. However, the value *p* = 0.0623 shows that statistical significance was not achieved at the commonly used 5% significance level. In other words, we can assume greater material deflection with the RsO pattern, but the result cannot be interpreted as clearly confirmed.

The analysis of the influence of the sawing pattern on twist warping revealed that timber produced using the RsO sawing pattern exhibited lower twist warping values compared to the RsP variant. The correlation coefficient r = −0.3424 indicates a slight negative correlation between the sawing pattern and the amount of twisted warping, but the results were not statistically significant (*p* = 0.1395). Despite this, visual analysis suggests a potential reduction in deformations when using the RsO sawing, which could be relevant for the optimization of sawing patterns.

A comparison of several literary sources [11,14,18] shows that the dimensional stability of sawn timber is significantly affected by the chosen sawing pattern. Studies by [11,18] confirm that the type of sawn determines the distribution of internal growth stresses as well as the subsequent amount of warping after sawing or drying process. Research by [14], which examined different methods of sawing *Cryptomeria japonica* (Sugi) with a diameter of 30 to 40 cm, found that larger diameters (from 40 cm) reduce the degree of warping as they better distribute internal stresses.

### 3.4. Evaluation of the Effect of the Original Position in the Log on the Dimensional Stability of Beech Timber

The dimensional stability of wood is a key parameter in evaluating the quality of timber, especially when used in construction applications. A non-contact method of optical deformation measurement using high-speed cameras and ARAMIS 3D software was used to accurately monitor these changes. This system enabled three-dimensional monitoring of surface displacements in real time with high accuracy.

The influence of the original position in the log on the dimensional stability of beech timber was evaluated using multiple regression analysis. In the evaluation, we differentiated between two positions of the timber, namely close to the bark (1) or outside the bark (2). The zone near the pith, or the influence of this original position in the log, was eliminated by changing the sawn itself.

**Figure 10:**
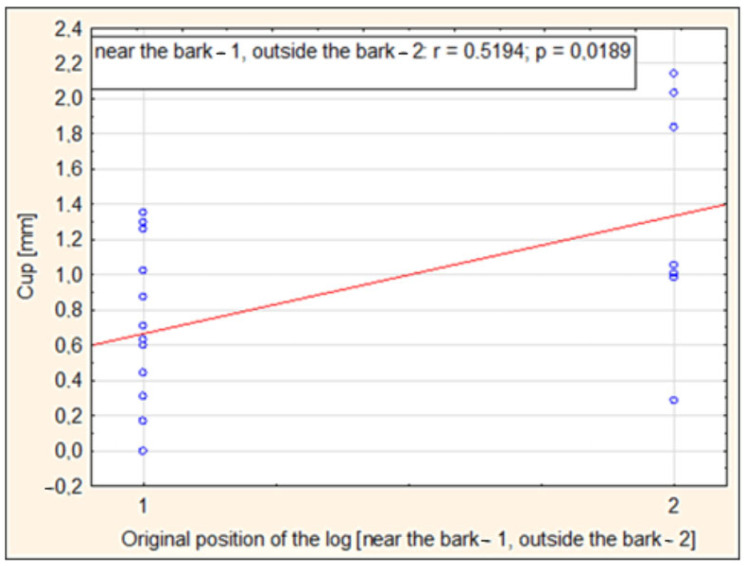
Dependence of cup warping on the original position in the log (near the bark—1, outside the bark—2).

The results of the analysis of the influence of the original position of the log in the cut on the occurrence of cup warping showed that timbers originating from the area closer to the center of the log exhibited a higher degree of this warping compared to timbers sawn near the bark (Figure 10). Pearson’s correlation coefficient (r = 0.5194) indicates a moderately strong positive correlation between these variables, and the value *p* = 0.0189 confirms the statistical significance of this relationship. These findings suggest that the location of the log in the trunk can significantly affect the shape stability and intensity of cup warping in the final product.

The correlation between the original position of the sawn timber in the log and the twist warping of the sawn timbers did not show a statistically significant dependence. Despite the moderate trend indicated by the regression equation (Twist [mm] = 11.8371 + 2.1529·x), the correlation coefficient (r = 0.0722) indicates a practically zero correlation, and the value *p* = 0.7622 confirms that the difference between the individual groups is not significant. For this reason, it can be concluded that the position of the log (at the bark or outside the bark) has no significant effect on the formation of spiral warping during the artificial drying process.

**Figure 11:**
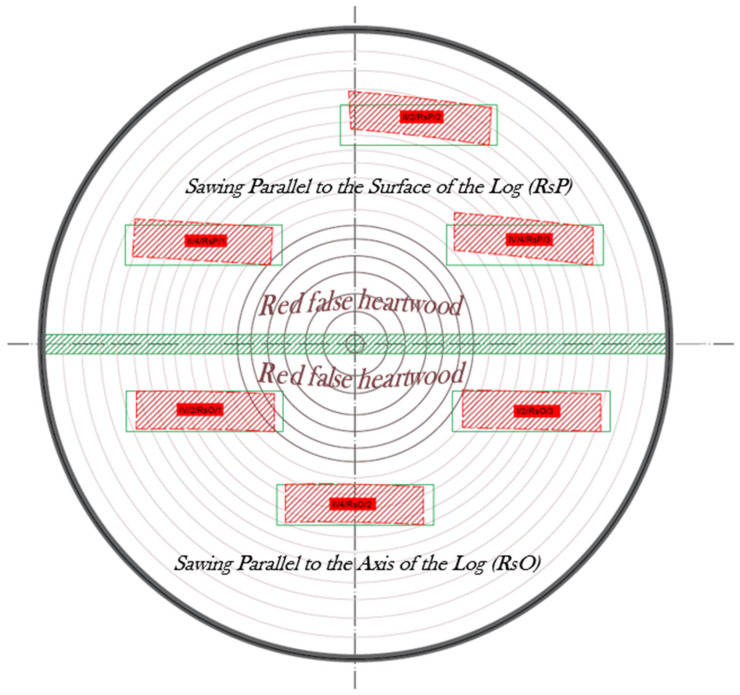
Sawing pattern showing the dimensional stability of beech lumber in terms of the resulting cup, and twist warping before (green line) and after the drying process (red line).

The results were also processed into a representative sawing pattern, which shows the differences in the dimensional stability of beech sawn timber from the perspective of the sawn pattern used: parallel to the surface of the log (RsP) and parallel to the axis of the log (RsO) (Figure 11).

It was found that the sawing pattern parallel to the surface of the log (RsP) produced more stable timber in terms of cup warping (0.9 mm). Conversely, in terms of twist warping, the sawing pattern parallel to the log axis (2.64 mm) produced better results. With the RsP cut, the twist warping was at an average value of 5.77 mm, which indicates that the RsP cut produced timber more predisposed to twist warping. The average value of cup warping with the RsO log was 1.3 mm.

The reason for this behavior is due to the wood’s natural anisotropy. Sawing parallel to the surface (RsP) disrupted the internal structure of the material (releasing internal stresses), which probably contributed to increased dimensional instability, especially in the form of twist warping. In the second method, RsO, we found better overall stability of beech timber, which only confirms our claims. Overall, this was also aided by the type of timber produced, which consisted largely of radial timber (annual rings inclined at 90°), which, as we know and as was confirmed in this research, has a positive effect on the resulting stability. The results, therefore, show that sawing parallel to the axis produces more dimensionally stable timber.

According to [14], the warping of sawn timber after sawing changes significantly depending on the distance of the timber from the pith. Pith-containing timber exhibits the highest warping values, especially in smaller log diameters, because internal growth stresses are concentrated in this area. Conversely, timbers from the outer parts of the stem, where the growth rings are more parallel, have better dimensional stability. The findings of [13] confirm these findings and add that the position of the pith within the log is a decisive factor in the warping of the timber. Timbers sawn so that the pith is close to the center of the cross-section showed the greatest warping, while timbers completely outside the core retained higher dimensional stability. The authors also point out that the microfibril angle (MFA) gradient around the pith causes different longitudinal shrinkage, which is the primary cause of, for example, twist warping.

## 4. Conclusions

- There is a negative relationship between the slope of annual rings and cup warping. This means that a higher slope of annual rings improves the shape stability of beech timber, thereby contributing to higher dimensional stability.
- The relationship between the sawing pattern (sawing parallel to the surface of the cut—RsP; sawing parallel to the axis of the cut—RsO) and the measured cup warping proved to be slightly positive (r = 0.4242, *p* = 0.0623). This result suggests that these sawing patterns may have affected the resulting cup warping.
- When analyzing the effect of the original position in the log, a stronger correlation was observed near the bark and outside the bark (cutout perimeter) (r = 0.5194, *p* = 0.0189). This result suggests that the original position in the log has a more significant effect on the magnitude of cup warping. Timber manipulated closer to the bark (to the perimeter of the log) shows slightly greater warping, which is probably due to differences in the structure (different fiber cuts).
- Based on the representative sawing pattern created, it can be seen that the RsP cut produced less stable timber, which was mainly characterized by a high degree of twist warping. On the other hand, sawing parallel to the axis of the cut produced more stable beech lumber, where only minimal cup warping was evident. This shows that sawing parallel to the axis of the cut does not disrupt the structure (fiber cross-cutting) to such an extent that it causes significant shape instability.

These conclusions can be used to optimize the processing of beech sawn timber in industry, which will contribute to better quality of final products and minimize material losses.

Based on our results, for practical applications, it is recommended to produce beech timber with a higher slope of annual rings to improve dimensional and shape stability. Sawing parallel to the axis of the log (RsO) should be preferred, as it produces more stable lumber with minimal cup warping, while sawing parallel to the cut surface (RsP) may increase twisting and shape instability. Attention should also be paid to the original position of the timber in the log, as boards cut closer to the bark tend to exhibit greater warp twist.

## Figures and Tables

**Table 1:** Level of risk of taper on the stability of beech lumber [8].

Taper cm/m	Our Results (Taper)				Taper/Risk	Impact
	Log					
	1	2	3	4		
0–8.7	0.70	1.35	1.95	0.71	Safe	Normal tapering, minimal impact on the strength and stability of lumber
8.7–17.6	-	-	-	-	Mildly	Localized reduction in the strength, stiffness, and stability of lumber
17.6–26.8	-	-	-	-	Significant	Significant decline in the mechanical properties and stability of lumber
> 26.8	-	-	-	-	Dangerous	Risk of failure, unsuitable for structural timber due to instability

**Table 2:** Results from measuring the slope of annual rings, cup, and twist warping on beech timber.

Sample	Slope of Annual Increment [°]	Cup [mm]	Twist [mm]	Type of Lumber
I/2/RsP/2	10.8	1.01	16.68	T
I/4/RsP/1	54.5	0.00	6.22	R
I/4/RsP/3	34.0	0.63	7.72	T
I/2/RsO/3	75.0	0.71	6.64	R
I/4/RsO/2	16.1	1.84	10.64	T
II/2/RsP/1	35.5	0.00	6.38	T
II/2/RsP/2	24.0	0.98	38.85	T
II/4/RsP/1	68.7	0.17	4.54	R
II/2/RsO/2	0.05	2.14	9.71	T
II/4/RsO/2	0.08	1.05	6.65	T
III/2/RsP/2	21.3	1.02	12.15	T
III/4/RsP/1	58.7	1.35	12.70	R
III/2/RsO/1	66.3	1.30	6.32	R
III/2/RsO/2	69.5	0.31	6.57	R
III/4/RsO/2	32.0	2.03	12.77	T
IV/2/RsP/1	15.1	1.26	67.48	T
IV/2/RsP/2	21.9	0.29	17.70	T
IV/4/RsP/3	57.5	0.60	20.18	R
IV/2/RsO/1	82.0	0.87	10.96	R
IV/4/RsO/3	50.5	0.44	14.01	T

Notes: 8—radial lumber, 12—tangential lumber; T—tangential lumber and R—radial lumber. (I) log number/(2) layer number/(RsP) sawing pattern method/(2) order of produced lumber in the layer.

## Data Availability

The data presented in this study are available on request from the corresponding author.
